# European Court of Justice delivers no justice to Europe on genome‐edited crops

**DOI:** 10.1111/pbi.13200

**Published:** 2019-07-22

**Authors:** Alan H. Schulman, Kirsi‐Marja Oksman‐Caldentey, Teemu H. Teeri

**Affiliations:** ^1^ Production Systems Natural Resources Institute Finland (Luke) Helsinki Finland; ^2^ Institute of Biotechnology University of Helsinki Helsinki Finland; ^3^ Viikki Plant Science Centre Helsinki Finland; ^4^ VTT Technical Research Centre of Finland Ltd. Espoo Finland; ^5^ Department of Agricultural Sciences University of Helsinki Helsinki Finland

**Keywords:** genome editing, GMO regulations, NBT, new breeding techniques, CRISPR/Cas

## Genetic variation is natural and needed for crop improvement

The advent of agriculture about ten millennia ago, the Green Revolution of the 1960s, and all agriculture in‐between and since were founded on identification and use of genetic variation. Traditional farmers selected higher producing or better tasting variants and propagated them. The 19th‐century advent of plant breeding exploited variation by use of sexual crosses. The science of breeding made great progress through the application of Mendelian, quantitative, and population genetics, heterosis, and ultimately molecular markers and genomic selection. However, modern breeders in essence still search for the variation that gives needed traits and introduce it into their breeding programmes. The rest is just combining alleles.

However, there is only finite variation within our crop species, and genetic incompatibility limits the amount that can be introgressed from outside the species. Moreover, wide crosses with exotic germplasm can bring, together with the desired trait such as disease resistance, many undesired traits such as seed shattering or low yield, which had been earlier painstakingly bred out of the elite parent. After LJ Stadler demonstrated the use of X‐rays to mutagenize barley and maize (Stadler, 1930), breeders began to create their own variation, using random mutagenesis followed by selection, called ‘mutation breeding’. At the beginning of 2019, the joint FAO/IAEA mutant variety database (mvd.iaea.org/) contained 3284 plant varieties released in more than 60 countries, which were either the direct products of mutagenesis or their progeny. These span at least 214 plant species, including not only the major cereals and grain legumes, but also oil crops, fibre crops, herbs, fruits and ornamentals. Prominent examples include the following: the rice varieties Amaroo (Australia), Zhefu 802 (China), RD6 and RD15 (Thailand); the malting barley varieties Diamant and Golden Promise; NIAB‐78 cotton (Pakistan); Rio Star grapefruit (USA).

## Gene editing as a response to the off‐target problem of mutagenesis

Chemical mutagens as used in mutation breeding induce mutations at a frequency ranging from once every 24 kb to 1000 kb (Spencer‐Lopes *et al*., 2018). For example, EMS‐induced mutagenesis in common (hexaploid) wheat induced 104 779 SNPs throughout the genome (Hussain *et al*., 2018). While mutagenized populations are good platforms for reverse genetics (TILLING; Acevedo‐Garcia *et al*., 2017), many back‐crosses are required to purify a specific desired mutation away from the mutagenized background. In the last few years, genome editing, a set of highly accurate tools for introducing specific genetic variations, has been taken into use worldwide. CRISPR/*Cas9* is perhaps the best‐known and most widely adopted example of those tools (Hilscher *et al*., 2017). The development of genome editing methods has been widely celebrated in the scientific community for several reasons. As a research tool, they offer an efficient platform for analysis of gene function through reverse genetics. Moreover, they offer a means of knocking out a gene whose function is known, in order to alter an associated crop trait (Yin *et al*., 2017). Recently, the advent of base editing makes possible the tweaking of gene function, in essence through the creation of targeted allelic variation (Kim, 2018).

The issue of off‐site mutations by CRISPR/*Cas9*, which together with a lack of targeting is also the major drawback of chemical‐ and radiation‐based mutagenesis, has been given attention by researchers. Editing experiments indicate that off‐site mutations are extremely rare or undetectable (Feng *et al*., 2018; Lee *et al*., 2019), even if potential sites can be identified by software. Given the low level of off‐site mutations, back‐crossing of the T0 generation will eliminate the secondary mutations with high efficiency, unlike for conventionally mutagenized lines.

### Allowing fishing by dynamite but forbidding fish hooks and lures

Given the highly accurate nature of CRISPR/*Cas9* ‐mediated editing compared to conventional mutagenesis, why did the ECJ take a *laissez faire* approach to varieties produced with the latter, but subject genome‐edited varieties to onerous regulation as a GMOs? The ruling is equivalent to allowing fishing by dynamite but forbidding fish hooks and lures. Although the *Cas9* and guide RNA (gRNA) construct is often transformed into plants during the editing process, these can be segregated away in the T1 and subsequent generations. Moreover, for vegetatively propagated crops and perennials, transgenesis‐free methods have been developed (Danilo *et al*., 2019). To understand why the ECJ nevertheless regards edited plants as GMOs, regardless of the presence of the construct, it is useful to look at the ruling. The judgement of 25 July 2018 (Case C‐528/16; http://curia.europa.eu/juris/documents.jsf?num=c-528/162018) cites Directive 2001/18 (https://eur-lex.europa.eu/legal-content/EN/ALL/?uri=celex%3A32001L0018), which regulates GMOs, and applies the ‘precautionary principle’ for new approaches. However, 2001/18 excludes under its Annex 1B ‘certain techniques of genetic modification which…have a long safety record’ and do not involve recombinant DNA, in particular mutagenesis.

So how did genome editing as a mutagenesis method fall afoul of the Court? The trouble appears to be a linguistic and logical tangle over ‘modification’. Under 2001/18, a GMO is an organism whose DNA has been altered ‘in a way that does not occur naturally’. ‘Mutagenesis’ is excluded under 2001/18 if not involving the use of a ‘genetically modified organism’. Now, the Court finds that the editing techniques in particular alter DNA in ‘a way that does not occur naturally’ and therefore generate GMOs. This is because while Annex 1 A and Annex 1 B of 2001/18 include only recombinant methods *in vitro* or *in vivo* as well as cell fusion as making GMOs, they do not explicitly exclude mutagenesis because it is not included in the list *not* making GMOs (*e.g*., *in vitro* fertilization, polyploidy induction). Moreover, 2001/18 held mutagenesis as a ‘technique of genetic modification’, even if not leading to a GMO. So the Curia uses the original confusions in 2001/18, whereby DNA can be modified but not result in a genetically modified organism under law if the method used had been ‘conventional’ with a ‘long safety record’. It holds that the risks of new mutagenesis techniques possibly may be the same as those of transgenesis, that the alterations are ‘unnatural’, so therefore the precautionary principle holds and the new methods must be regulated like transgenesis. Thus, the judges, ignoring the science, forced genome editing under the outmoded Directive 2001/18.

The idea that a technique, which uses a process found in nature, is ‘unnatural’ is illogical. The idea that a single mutation could pose risks, which the same mutation mixed in with a thousand others does not pose, is nonsensical. The older, scattershot methods introduce random changes throughout the DNA, many of which remain in the final variety placed on the market and have undetermined effects. In contrast, genome editing is highly accurate and can only be undertaken with precise knowledge of the target gene. Thus, it is a wonderment that the ECJ ignored the statements of EU advice bodies, both the Scientific Advice Mechanism (SAM) of the European Commission and the European Food Safety Agency (EFSA), which held edited plants to be equivalent to those produced by conventional means.

In contrast to the final EJC view, the preliminary ruling of EU Advocate General Michal Bobek (http://curia.europa.eu/juris/document/document.jsf?text=&docxml:id=198532&pageIndex=0&docxml:lang=EN) did suggest a channel by which genome‐edited plants might be outside the regulatory framework but nevertheless GMOs. His opinion is based on two principles: first, triggering of the precautionary principle must be based on broad scientific data and not merely fear of risk; second, the definition of mutagenesis cannot be fixed to its meaning at the turn of the millennium, just as that of ‘vehicle’ or ‘means of communication’ cannot be restricted to their sense of two centuries ago, but rather be adjusted to include newer approaches. Hence, like conventional mutagenized lines, edited lines would outside the regulatory framework of 2001/18, however, with the reservations that new methods must ‘…not involve the use of recombinant nucleic acid molecules or GMOs’. These caveats are very unclear, because Bobek defines neither ‘involvement’ nor ‘recombinant’. His view appears to differ from 2001/18 itself (Annex 1A Part 1(1)), whereby the recombinant nucleic acids in regulated GMOs must be ‘incorporated’ where they ‘do not naturally occur’ and be ‘capable of continued propagation’. In any case, the EJC finally maintained a premodern view of mutagenesis.

### Response to the ECJ ruling

The response to the ruling among the worldwide scientific community has been universally excoriating. The European Plant Science Organisation (EPSO), representing 200 research institutions from 30 countries and over 26 000 people working in plant science, expressed disappointment and noted the lost opportunities for Europe. A consortium of 116 European research institutions spearheaded by VIB/University of Ghent sent an open letter to EC President Jean‐Claude Juncker detailing their deep concern over the downsides. A coalition of 13 countries from around the world issued a statement at an autumn 2018 WTO meeting, supporting policies that enable innovation by genome editing.

### Consequences of the ruling

European Parliament and Council Directives need to be ‘transposed’, that is, implemented in national law, for which the ECJ ruling will cause no end of trouble. The ruling will be impossible to enforce because edited plant varieties are indistinguishable from ones derived from chemical or radiation mutagenesis or from crosses to exotic germplasm. So Member States will find it difficult to enforce laws based on Directive 2015/412, the extension of 2001/18 that allows them to prohibit cultivation of genetically modified crops on part or all of their territories. Two cultivars with identical nucleotide changes, one made by editing and the other not, neither with exogenous DNA, will need to be separately regulated under the law. Foods that contain more than 0.9% GMOs should be labelled for sale in the EU; however, edited ingredients will not be identifiable by their DNA. Foreign producers in countries not regulating genome‐edited crops as GMOs (Table 1) have no legal responsibility to track or label them; either all food products from such sources will need to be banned, or the ruling will be meaningless. This has the potential to raise serious international trade issues.

**Table 1:** Current regulatory status of plant genome editing for selected countries outside the EU

Region/country	Current genome editing status	Example products in market pipeline
North America		
Canada	Product‐, not technology‐based.	
USA	Not GMO	At least 20 products, including high‐oleic‐acid soy oil; high‐fibre wheat; alfalfa; cold‐storable potato; reduced‐browning potato; coeliac‐friendly wheat; maize with waxy starch
South America		
Argentina	Not GMO	At least 10 pending plant varieties
Brazil	Not GMO	Cacao
Chile	Not GMO	
Columbia	Not GMO	Micro‐Tom tomatoes
Honduras	Not GMO	
Paraguay	Not GMO	
Uruguay	Expected to harmonize with other South American countries	
Other		
Australia	Editing without template not regulated as GMO	
Israel	Not GMO if no transgene	
Japan	Not GMO	
New Zealand	GMO	
Norway	Not regulated as GMO if change can occur by conventional methods	
Philippines	Not GMO	
Russia	New decree exempts GE crops from GM regulations	
Switzerland	Draft revision to GMO law expected 2019	

The decision by the ECJ is moreover simply bad policy for Europe. Big agribusiness has the expertise and deep pockets to overcome the regulatory hurdles of GMO legislation. In fact, the judgement gives them an open playing field by restricting the entry of small players, such as many breeders in Europe. Permission only to import a GMO on average can cost €11 m–€16.7 m and take 6 years (https://www.europabio.org/agricultural-biotech/faq/gmos-and-the-european-union/how-long-does-it-take-gm-crop-import-be-approved-and-how-much-does-cost). Given that at least 15 gene‐edited plants, most from small players, had been developed by 2018 in the United States alone (https://www.theparliamentmagazine.eu/articles/partner_article/europabio/how-europe-has-priced-out-innovation-example-plants), solutions to make crops more sustainable, healthy, healthful and productive will be sought outside Europe. Statements by several large breeders indicate that they will either breed edited crops for markets outside Europe, move their editing research from Europe, or both (https://european-biotechnology.com/up-to-date/backgrounds-stories/story/cjeu-ruling-triggers-exodus-of-eu-plant-research.html). The growing list of countries (Table 1) that have either excluded genome‐edited varieties from GMO regulations or that implement product‐ and not process‐based regulations grows by the month, leaving the EU increasingly isolated. Moreover, the paperwork and delays imposed on academic research and field trials will have a chilling effect, driving talent and innovation from Europe. In sum, novel crops will be developed for growing conditions outside of Europe by breeders outside Europe, with research and investment likewise directed elsewhere. European food producers will fail to receive locally sourced raw ingredients with improved and novel qualities to meet public needs.

### How forward?

Europe and the rest of the world face enormous agricultural challenges. Meeting sustainable development goals for a population of 9.7 billion projected for 2050 with less fertilizer, a fixed water budget, on less land, and under a changing climate will require novel cultivars as rapidly as possible. Genome editing is a green solution, one of many tools that plant scientists, breeders, and farmers desperately need now. As the 116‐institution letter to Juncker stated and EPSO likewise holds, the next Commission must urgently prioritize the matter, and plants with small genetic changes and no foreign genes must be outside of the regulatory regime. Ultimately, EU GMO regulations urgently need updating to a product‐based and not method‐based system. Current mutation‐derived crops are cultivated on tens of millions of hectares. Likewise, edited varieties with targeted, knowledge‐based changes can help to provide a secure, economically and environmentally sustainable food supply to all the world, should the regulatory authorities choose to rely on evidence for their decisions. Such crops are appearing outside Europe already; it is time for Europe to bring the benefits of its research investment in the plant sciences home.

## Author contributions

AHS conceived and wrote the manuscript; K‐M.O‐C and T.H.T. contributed to the concept as well as to the writing of manuscript and to critical revisions. All authors read and approved the final manuscript.

## Conflict of interest

The authors declare that they have no competing financial interests.
